# Notes

**Published:** 1896-05

**Authors:** 


### Notes.

The Sixth Annual Meeting of the Confederation of State Examining Boards will be held in Room No. 1, Hotel Aragon, Atlanta, Ga., (which offers reduced rates), Monday, May 4, 1896, at 10 o'clock a. m.

The following programme will be carried out, namely:

1. Introductory remarks by the Vice-President.
2. Report of the Committee on revision of Constitution and By-Laws.
3. Discussion and action thereon.
4. Report of the Secretary.
5. Annual Address of the President.
6. Address, by James Russell Parsons, Jr., Albany, Director of Examinations, University of the State of New York. Preliminary Education, Training and Practice in New York.
7. Paper—The limitations of the Standard of Modern Educational Requirements, as Determined by State Medical Examining Boards, by Joseph M. Mathews, M. D., Louisville, Ky.
8. Paper—(Subject to be announced,) by William S. Foster, M. D., Pittsburg, Pa.
9. Some obstacles to an Inter-State recognition of a State license to practice medicine, with suggestions for their removal, by Charles McIntire, M. D., Easton. Pa.
10. Miscellaneous Business.
11. Election of Officers.

The objects of the Confederation, though purely of an advisory nature, are to discuss questions that pertain to State licensure in medicine, with a view to a comparison and improvement of methods, a collection and dissemination of information on the subject, and to consider any and all propositions that have for their purpose the advancement of the standard of medical education in the United States.

The Officers of the Confederation, therefore, beg to extend a cordial invitation to members of State Examining Boards, and all ex-members of State Boards, and especially to every physician and educator who is friendly to the objects sought, to attend the meeting and participate in the proceedings.

Please indicate by a note to the Secretary, whether you will attend.

By order of the Executive Council.

B. M. Griffith, Secretary.

THE local committee of arrangement of the American Medical Association is busily at work collecting the necessary funds. Private citizens are giving generously and the support of the physicians is counted upon. The Atlanta Society of Medicine contributed $200 from its surplus.

The programmes are being printed and show a list of extremely interesting papers by well-known men from all over the country.

The various sections have been suitably provided for, the smaller ones at rooms in the Kimball and Aragon hotels, and the larger at the Y. M. C. A. Halls. There will be a barbecue at Lithia Springs on Wednesday, May 6, and a general reception at the Capital City Club, Thursday, May 7.

About fifteen hundred doctors will be present, and there is every promise of a full and profitable meeting.

---

THE *Fort Wayne Medical Journal* rises to protest against the wholesale destruction of germs which is going on and seems fearful that the race will go the way of the buffalo and the great auk.

If the people of Indiana are suffering for lack of germs Atlanta can furnish them assorted lots of typhoid and tubercle bacilli, under special warranty of excellence. We, in this section, are not, as yet, apprehensive of a germ famine.

---

THE honor men of the graduating class of the Southern Medical College, were: First honor, R. L. Whipple; second honor, E. L. Awtry; third honor, Lucien Lofton.

Of the Atlanta Medical College, Dr. K. L. Reid, of Canada received the first honor. The second honor was divided between Ned C. Berry, of Florida, and J. L. Snider, of Georgia.

---

ONE of the best papers read at the meeting at Augusta, was that of Dr. J. B. Morgan on "Colles' Fracture," which we shall reproduce shortly in these columns.

---

DR. THEODORE LAMB, of Augusta, died there April 15. Dr. Lamb was one of the ablest and most popular members of the profession of Augusta.

